# Diagnosis, Prevention, and Treatment of Radiotherapy-Induced Xerostomia: A Review

**DOI:** 10.1155/2022/7802334

**Published:** 2022-08-27

**Authors:** Yanli Li, Xuehan Li, Runxuan Pang, Guang Yang, Mingxu Tian, Tengyu Zhao, Yunhan Sun, Eui-Seok Lee, Heng Bo Jiang, Jianmin Han

**Affiliations:** ^1^Department of Dental Materials, Peking University School and Hospital of Stomatology, National Center of Stomatology, National Clinical Research Center for Oral Diseases, National Engineering Laboratory for Digital and Material Technology of Stomatology, Beijing Key Laboratory of Digital Stomatology, Research Center of Engineering and Technology for Computerized Dentistry Ministry of Health, NMPA Key Laboratory for Dental Materials, Beijing 100081, China; ^2^The Conversationalist Club, School of Stomatology, Shandong First Medical University and Shandong Academy of Medical Sciences, Jinan, Shandong 250117, China; ^3^Department of Oral and Maxillofacial Surgery, Graduate School of Clinical Dentistry, Korea University, Seoul 08308, Republic of Korea

## Abstract

In patients with head and neck cancer, irradiation (IR)-sensitive salivary gland (SG) tissue is highly prone to damage during radiotherapy (RT). This leads to SG hypofunction and xerostomia. Xerostomia is defined as the subjective complaint of dry mouth, which can cause other symptoms and adversely affect the quality of life. In recent years, diagnostic techniques have constantly improved with the emergence of more reliable and valid questionnaires as well as more accurate equipment for saliva flow rate measurement and imaging methods. Preventive measures such as the antioxidant MitoTEMPO, botulinum toxin (BoNT), and growth factors have been successfully applied in animal experiments, resulting in positive outcomes. Interventions, such as the new delivery methods of pilocarpine, edible saliva substitutes, acupuncture and electrical stimulation, gene transfer, and stem cell transplantation, have shown potential to alleviate or restore xerostomia in patients. The review summarizes the existing and new diagnostic methods for xerostomia, along with current and potential strategies for reducing IR-induced damage to SG function. We also aim to provide guidance on the advantages and disadvantages of the diagnostic methods. Additionally, most prevention and treatment methods remain in the stage of animal experiments, suggesting a need for further clinical research, among which we believe that antioxidants, gene transfer, and stem cell transplantation have broad prospects.

## 1. Introduction

Head and neck cancer is defined as a localized malignant tumor of the head and neck. A commonly used treatment regime includes surgery combined with RT [1]. Despite the continuous progress in RT techniques, damage to the surrounding healthy cells or tissues is possible. The SGs proliferate slowly and are sensitive to IR; therefore, damage to SGs is common and irreversible [2–4]. The potential mechanism of IR leading to the loss of SG function has been studied in animal models, which may be related to DNA damage, loss of acinar cell number, increase in reactive oxygen species (ROS), decrease in proliferation and differentiation ability of stem/progenitor cells, and abnormal calcium signaling. In later stages, it may be associated with changes in blood vessels, glandular fibrosis, and inflammation [2, 5, 6].

SG dysfunction can lead to reduced salivary secretion, resulting in xerostomia, which significantly affects the quality of life of patients [1]. Saliva, although more than 99% water, contains many important functional components [7]. The proteins in saliva include mucin-, immunoglobulin-, and proline-rich proteins, which play vital roles in lubrication, antibacterial activity, defense, and protection of the teeth [8–10]. Additionally, other components of saliva can help digest food, regulate pH, strengthen the sense of taste, neutralize harmful substances, and promote wound healing [11]. Therefore, if SG function is damaged, leading to xerostomia, it will inevitably have an impact on the whole mouth, causing oral dryness, mucosal atrophy and ulcer, mastication and swallowing difficulties, caries, infection, and other serious consequences [1, 12].

A MEDLINE/PubMed search was conducted using the terms “xerostomia” OR, “salivary gland hypofunction” AND, “diagnosis” OR, “treatment” OR, “prevention” OR, “questionnaire” OR, “saliva flow rate measurement and oral moisture-checking device” OR, “imaging techniques” OR, “advances in RT” OR, “antioxidants” OR, “botulinum toxin” OR, “submandibular gland transfer” OR, “growth factors” OR, “rapamycin and limonene” OR, “saliva substitutes” OR, “pharmacological salivation agents” OR, “acupuncture and electrical stimulation” OR, “hyperbaric oxygen therapy” OR, “gene therapy” OR, “stem cells.” Published articles from 2010 to 2022 were included, and some significant references were also reviewed. After the initial search, literature with incomplete data and low credibility were excluded. Since many of the methods in the prevention and treatment parts mentioned in this article still lack sufficient clinical trials to prove their effectiveness, we have included some results from animal experiments to demonstrate the potential value of the methods.

This article discusses the xerostomia diagnostic methods that are currently being used and those being newly developed, as well as the current and potential strategies for SG recovery and presents their advantages and disadvantages. The primary aim is to provide guidance for further research in this field.

## 2. Diagnosis

### 2.1. Questionnaire

Questionnaires, which are not limited to the assessment of dry mouth but also cover other complications, play a significant role in evaluating xerostomia. They are low-cost, easy to complete by patients and can be evaluated by clinicians. This method can also be used for long-term detection. However, the questionnaires are subjective; therefore, they cannot always reflect the SG function [13, 14].

The Xerostomia Questionnaire, Xerostomia Inventory, Summated Xerostomia Inventory, and visual analog scale are commonly used to evaluate xerostomia, and their effectiveness has been confirmed [15–18]. In addition, the Groningen RT-Induced Xerostomia questionnaire, which was developed in 2010, was used to evaluate the degree of xerostomia and salivary viscosity and is the only tool explicitly developed in the RT-induced xerostomia population [14, 19]. The Groningen RT-Induced Xerostomia questionnaire contains 14 items that are measured during the daytime and nighttime, allowing it to distinguish between a patient's xerostomia in different time frames [19]. It has good reliability, responsiveness, and criterion validity, but Assas et al. found that the construct validity of the questionnaire was indeterminate [14, 19]. The Multidisciplinary Salivary Gland Society questionnaire was created in 2021. It mainly quantified the symptoms of xerostomia, including 20 questions and two scoring systems (Q3 for question answering and Q10 for the visual analog scale). The reliability coefficients of both scoring systems were ≥0.9; however, more clinical trials are needed to further validate the questionnaire. The developers of the MGSG questionnaire recommend using the Q10 system because it is easier for patients to understand and more accurate in translating into different languages [20].

### 2.2. Saliva Flow Rate Measurement and Oral Moisture-Checking Device

Many studies have shown a moderate correlation between saliva flow rate and xerostomia, so it can also be an indicator of xerostomia. In addition, the saliva flow rate measurement can better reflect the SG function, which is more accurate and reliable than the questionnaires in the evaluation of SG function [21–23]. For some patients, measuring unstimulated whole saliva (UWS), stimulated whole saliva (SWS), or the saliva of a single SG or minor SGs is essential for a clinical diagnosis [24]. The current and new methods for measuring saliva flow rates are summarized in Table 1.

An oral moisture-checking device is used to diagnose xerostomia by measuring the oral moisture of the lingual and buccal mucosa [37]. The results of oral moisture measurements have a weak positive correlation with saliva flow rate, possibly because the chief complaint of xerostomia is not always correlated with saliva flow rate [37]. Third-generation oral moisture-checking devices have been widely used in xerostomia diagnosis. Furthermore, Fukushima et al. first confirmed the effectiveness of a fourth-generation device and its reliability in all age groups. However, the force of the instrument sensor placed on the oral mucosa is difficult to control [37, 38]. In conclusion, the oral moisture-checking device appears to have excellent prospects.

### 2.3. Imaging Techniques

RT-induced gland atrophy and necrosis can lead to pathological findings such as reduced volume, increased heterogeneity, and unclear boundaries of the gland, which can evaluate SG function and then predict xerostomia [3]. Contrast-enhanced computed tomography with high-density structural and spatial resolution can quickly examine the appearance and cysts of the SGs with ease. However, it has certain ionizing radiation; therefore, ultrasound imaging and magnetic resonance imaging (MRI) can be used to evaluate SG function in non-invasive conditions without ionizing radiation [3, 39]. Ultrasound imaging is used to evaluate gland function based on the information obtained from ultrasound scans, such as size, inflammation, and homogeneity [40]. It is cost-effective but does not adequately reveal lesions [39, 41]. MRI evaluates the degree of SG injury based on the decrease in volume and increase in signal intensity of T2-weighted images [42]. The MRI detection of SGs is highly sensitive, expensive, and heavily affected by metals [3, 42]. Salivary gland scintigraphy is an imaging technique used to measure the uptake and excretion of SGs using the radioactive tracer Technetium-99m pertechnetate [43]. Salivary gland scintigraphy has the advantages of being noninvasive, easy to perform, reproducible, and well tolerated by patients. However, the lack of accurate quantitative reference values and the absence of standardized protocols limit its widespread application [43, 44].

Sialography is another important imaging method the allows a better prediction of xerostomia by assessing the status of the salivary ducts [45]. Owing to the drawbacks of X-ray sialography, such as the injection of contrast agents, ionizing radiation, and the considerable risk of intubation failure, an advanced magnetic resonance (MR) sialography has been proposed [3, 46]. MR sialography uses saliva itself as the contrast medium, which enables the technique to observe changes in a small amount of saliva in the salivary ducts to effectively assess IR-induced xerostomia [46]. In general, MR sialography offers high security, high accuracy, and has the potential for development [3, 46].

## 3. Prevention

### 3.1. Advances in RT

Compared to traditional RT, intensity-modulated radiation therapy (IMRT) can adjust IR according to the shape of the target region. It can maximize the IR dose to tumors and reduce the dose that endangers normal tissues and organs, thereby improving the therapeutic effect [47]. Several studies have found that IMRT can efficiently preserve or restore SG function better than conventional RT [47, 48]. Analysis by Ge et al. also showed that the health condition and cognitive function were significantly better in patients in the IMRT group than in the conventional RT group [47]. Volumetric modulated arc therapy (VMAT) is a promising treatment technique [49] that significantly increases the number of beams, improves efficiency, and reduces the uncertainty of equipment [50, 51]. Compared with IMRT, VMAT has advantages such as dose sparing, improved uniformity, reduced IR range, and aiding in alleviating acute dysphagia [49, 52].

In recent years, intensity-modulated proton therapy (IMPT), an emerging and promising treatment for head and neck cancer, is showing reduced toxicity to healthy tissues compared to IMRT and VMAT [53, 54]. This is because the Bragg peak phenomenon of proton beam therapy can generate a more favorable dose distribution curve than photon-based RT techniques [54]. However, IMPT is still challenged by uncertainty in the particle range and the difficulty of adapting to complex anatomical structures [53].

With the development of science and technology, new RT techniques have gradually replaced traditional RT techniques, providing excellent locoregional control and toxicity reduction [51]. However, owing to more professional operating techniques and expensive equipment, popularizing new RT techniques is difficult.

### 3.2. Antioxidants

**Figure 1:**
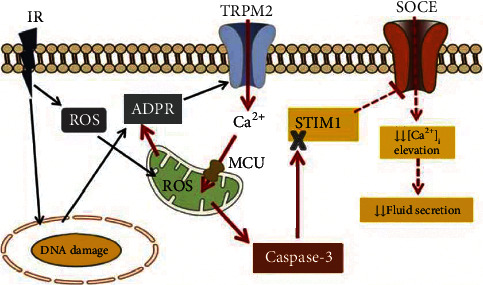
Model showing the early consequences of RT and mechanisms of persistent decrease in salivary secretion. Adenosine diphosphate ribose (ADPR) is the intracellular ligand that binds to and gates. TRPM2: transient receptor potential melastatin 2. MCU: mitochondrial Ca2+ uniporter. SOCE: Store-operated Ca2+ entry. STIM1: stromal interaction molecule 1, IR: irradiation. ROS: reactive oxygen species [55].

Maintaining a normal calcium concentration in acinar cells is a key factor in stimulating salivary secretion, but the presence of ROS will disrupt the normal transfer of intracellular calcium, as shown in Figure 1 [55]. Several experiments have shown that the initial level of ROS in SG cells increases after IR, and the following radioactive protective agents are able to scavenge oxygen free radicals and inhibit oxidative stress.

TEMPOL is a superoxide dismutase (SOD) analog [56, 57]. In mouse models, TEMPOL has been reported to protect against IR-induced SG injury [57, 58]. MitoTEMPO, as a mitochondria-targeted antioxidant, is similar to TEMPOL, but it contains lipophilic cationic triphenyl, which enables it to easily penetrate the lipid bilayer and accumulate in the mitochondria [59]. Thus, all TEMPOL nitrogen oxides can reduce IR-induced transient receptor potential melastatin-2 activation by scavenging H_2_O_2_ or ROS, inhibit caspase-3, prevent the decrease in stromal interaction molecule 1 protein, maintain the store-operated Ca^2+^ entry pathway of normal Ca^2+^ entry mechanism, and protect acinar cells and microvascular endothelial cells [56, 60]. It can also selectively protect normal cells from the harmful effects of IR without affecting the radiosensitivity of tumor cells [56, 58, 60]. This is because such substances may be rapidly converted to hydroxylamine in tumors [58]. However, these TEMPOL nitrogen oxides cannot resolve the quality of saliva, such as the decrease in lysozyme level [56].

Alpha-lipoic acid, a natural compound with strong antioxidant effects, can chelate metal ions, inhibit the formation of oxygen free radicals, and regenerate many antioxidants. SG cells can be protected by preserving the signals induced by parasympathetic innervation and releasing regeneration signals that promote cell proliferation. When the dose is sufficient, it can also radiosensitize tumor cells [4, 61]. Epigallocatechin 3-gallate (EGCG), a phenolic antioxidant, inhibits free radical chain reactions by capturing peroxide free radicals. It is superior to other catechins because of the six phenolic hydroxyl groups in its structure [62]. A certain dose of EGCG effectively prevented apoptosis in IR-injured epithelial cells and protects against oxidative stress and inflammatory cell infiltration [63, 64]. However, the absorption and oral bioavailability of EGCG are low, and its role in the physiological conditions of SGs after an injury has not been studied extensively [65].

Erythropoietin, an endogenous glycoprotein hormone, increases when IR damages SG microvessels, ischemia, and hypoxia. Recombinant human erythropoietin has been shown to balance SOD and ROS levels [56, 66]. Notably, recombinant human erythropoietin administration may also activate erythropoietin receptors in cancer cells, making them IR resistant [66]. The specific mechanism of erythropoietin function in the glands and methods to reduce its tumor-protective effect are aspects of future research. Cordycepin, also known as 3-deoxyadenosine, has been reported to clear ROS and inhibit mitochondrial damage [67–69]. It is known to promote the mRNA expression levels of alpha-amylase 1 and aquaporin-5. However, cordycepin can be rapidly deaminated by adenosine deaminase *in vivo*; currently, the only solution to its short half-life is to increase its dosage [69].

Amifostine is a broad-spectrum cell protector and the only US Food and Drug Administration-approved drug with a radiological protective effect [70]. Alkaline phosphatase levels were higher in normal tissues than in tumor cells. Amifostine is hydrolyzed to reactive sulfhydryl compounds (WR-1065) by alkaline phosphatase in normal tissues, which can also play a role in scavenging oxygen free radicals and protecting cellular substructures to selectively prevent injury caused by IR [70, 71]. Its tumor-protective effect is controversial, but there is currently no evidence that amifostine reduces the efficacy of RT [72, 73]. Systemic administration of amifostine has serious side effects such as acute cutaneous and mucosal toxicity, hypotension, hypocalcemia, and vomiting [70]. Retroductal cannulation and injection can bypass systemic circulation, provide direct glandular access, and be locally administered to the SGs, reducing hypotensive effects compared with intravenous administration [74].

### 3.3. Botulinum Toxin

BoNT has been shown to inhibit SNAREs involved in acetylcholine release at the neuroglandular junction and receptors involved in acinar cell granule exocytosis to prevent xerostomia. Therefore, it can temporarily atrophy SGs and reduce the number of granules secreted from acinar cells. This may make acinar cells significantly less sensitive to IR, which protects the SGs [75–77].

A study found that mice that received intraglandular injections of BoNT showed increased salivary flow rate, increased glandular weight, and decreased periductal fibrosis after RT compared to that in noninjected animals. This suggests that BoNT has an anti-inflammatory effect that can attenuate RT-induced periductal fibrosis and neutrophil infiltration [78]. In addition, studies have reported that BoNT is safe and effective in humans. It also has a radiosensitizing effect on tumors and can be effectively used in combination with RT [79, 80].

Notably, several studies have shown a positive effect of BoNT on the prevention of xerostomia, although the use of BoNT requires more clinical trials since most of the current studies are limited to animal experiments [81].

### 3.4. Submandibular Gland Transfer

Recent studies have demonstrated that transferring the submandibular gland to the submental space can effectively reduce IR damage to the SGs and prevent xerostomia. Seikaly et al. reported that submandibular gland transfer was better than oral pilocarpine in preventing xerostomia, leading to an improved quality of life [82, 83]. However, submandibular gland transfer also has complications caused by surgery and many contraindications. Additionally, the potential risk associated with this method of incorrectly interpreting submandibular gland images resulted in a higher IR dose to the submandibular gland [84–86]. In this regard, to reduce the impact of IR on the submandibular gland, transferring a submandibular gland to the patient's forearm during RT and re-transplanting the gland back to its original position after RT was proposed. This study showed that IR-induced SG hypofunction is reduced. However, forearm transfer could only serve as a potential preventive approach until further validation [87, 88].

### 3.5. Growth Factors

Although the mechanism of xerostomia recovery is still unclear, it is noteworthy that in addition to vascular and nerve recovery, the related molecular regulatory mechanisms have been discussed widely. Pathways such as Wnt/*β*-catenin, Hedgehog, PDGF-FGF, Chrm1/HB-EGF, and laminin/integrin are thought to play important roles in xerostomia recovery. The PDGF-FGF pathway explains the possible mechanism of interaction between epithelial cells and neural crest-derived mesenchymal stem cells. The Wnt/*β*-catenin pathway plays a key role in the formation of branching morphology. Transient activation of the Wnt/*β*-catenin pathway was observed to reduce SG injury caused by RT, which may be related to its role in inhibiting apoptosis and preservation of functional SGs cells. However, a specific explanation of the above mechanism is beyond the scope of this review [89–91].

RT-induced xerostomia is associated with p53-dependent apoptosis; therefore, a variety of growth factors are considered to prevent this injury [92]. In this review, various typical growth factors have been described. Insulin-like growth factor-1 has the potential to treat xerostomia as assessed in animal experiments which may be mediated through increased levels of sirtuin-1. Sirtuin-1 promotes DNA repair in cells and maintains the activation of protein atypical kinase C zeta, ultimately promoting inhibition of SG dysfunction and apoptosis by stimulating activation of the endogenous Akt pathway [93–96]. Similarly, keratinocyte growth factor-1, hepatocyte growth factor, and epidermal growth factor are also believed to inhibit apoptosis through the Akt pathway to protect SGs and can positively affect stem cell proliferation [97–99].

In addition, vascular endothelial growth factor is believed to improve blood flow in SGs and restore their function [100]. However, vascular endothelial growth factor treatment is mostly limited to animal experiments, and further research is needed to determine whether it can be applied in clinics for xerostomia treatment.

### 3.6. Rapamycin and Limonene

Rapamycin and rapalogue, CCI-799, can induce autophagy by inhibiting target mTOR complex 1 to maintain SG homeostasis [101–104]. CCI-799 can increase SOD expression to suppress excessive ROS, which may be related to autophagy. Furthermore, CCI-799 has been shown to restore salivary flow rate, increase amylase levels, and inhibit the compensatory proliferation of cells [59, 104, 105]. Therefore, rapamycin and CCI-799 have potential use in xerostomia.

The presence of aldehyde dehydrogenase 3A1 (ALDH3A1) prevented excessive aldehyde accumulation from damaging the SGs. RT led to a decrease in ALDH3A1, which further promoted acinar cell apoptosis and reduced spheroid formation [92, 106]. Saiki et al. identified an aldehyde dehydrogenase activator, limonene, to be safer and more acceptable than previous Alda89 (safrole) [106, 107]. It has been experimentally confirmed that limonene can decrease aldehyde levels and improve SG function without protecting the growth of tumors that also contain ALDH3A1 [106]. However, its application is limited owing to its higher doses [108].

## 4. Treatment

### 4.1. Saliva Substitutes

Saliva substitutes are one of the most effective measures to relieve xerostomia caused by RT and have antibacterial and preventive effects on tooth demineralization. However, they can only be retained for a short time in the oral cavity and may trigger allergic reactions in patients [109–112]. An edible saliva substitute like oral moisturizing jelly is noteworthy because it contains buffering agents, has a neutral pH, and can improve the swallowing ability of patients, in addition to relieving xerostomia. This resolves the concern regarding commercially available saliva substitutes not being recommended owing to the use of preservatives [109, 113]. Additionally, hyaluronic acid solutions at certain concentrations are similar to saliva in terms of viscosity, elastic modulus, and network structure. They exhibit antibacterial and antioxidant effects, making them a potential candidate for a saliva substitute [12, 114].

### 4.2. Pharmacological Salivation Agents

Pilocarpine is an imidazole-based alkaloid and as a typical muscarinic M3 receptor agonist, it can act on SGs to increase the saliva flow rate. It can also promote the supplementation of adenocytes, which may be due to the promotion of SOX2^+^ cell activity [1, 115]. Research is being conducted to develop targeted delivery methods to minimize the side effects of drugs. Malallah et al. believe that fast-disintegrating buccal tablets containing pilocarpine can be rapidly dissolved or decomposed orally. This method can stimulate SGs and attenuate the off-target effect of the drug, but evidence to prove its use in the clinic is lacking [116]. Another idea is the use of oral adhesives, which can improve the retention time of drugs at treatment sites. Chitosan and other substances used have shown adhesion, stability, and controlled slow release to the mucosa [117].

### 4.3. Acupuncture and Electrical Stimulation

Acupuncture, which uses extremely thin solid metal needles inserted into a suitable subcutaneous area, is a low-risk treatment that has been reported to boost salivary secretion [118]. The mechanism of acupuncture in the treatment of xerostomia remains unclear. However, there are two possible explanations. First, acupuncture stimulates the nervous system to produce neuropeptides that have nutritional and anti-inflammatory effects on SGs. Second, acupuncture has a direct effect on SG blood flow [119]. However, most studies on acupuncture have significant heterogeneity and low comparability [120].

Similar to acupuncture, electrical stimulation has been included in clinical studies as a treatment with fewer side effects. Electrical pulses can stimulate nerves and affect the SGs, such as transcutaneous electrical nerve stimulation which is believed to directly stimulate the auriculotemporal nerve [121]. Though these studies have revealed some positive effects, the evidence is insufficient. The instruments need to be refined in shape and material to provide the appropriate electrical impulse to activate the nerves [122, 123].

### 4.4. Hyperbaric Oxygen Therapy

Hyperbaric oxygen therapy (HBOT) has the ability to affect cytokine responses, induce local angiogenesis, and mobilize stem cells [124, 125] which suggests its potential in the treatment of SG dysfunction. HBOT has been shown to improve xerostomia, the sense of taste, and the swallowing ability of patients [126, 127]. However, most of these studies lack a sufficient sample size and appropriate control groups. The efficacy of treatment is controversial due to factors such as the placebo effect and patient adaptation to xerostomia [126, 128]. The optimal start time of HBOT after RT and the number of treatments still need further research [126, 128]. Moreover, HBOT is not widely accepted by patients because of its prohibitive cost and inability to fully restore SG function [127].

### 4.5. Gene Therapy

**Figure 2:**
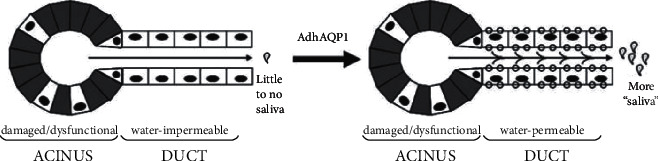
Schematic diagram of the mechanism of improving SG function after hAQP1 expression. Damaged SGs on the left and aquaporin-mediated SGs on the right. The gray acinar cells show they are damaged or dysfunctional by RT. The ductal epithelial cells with minimal damage by RT and surviving acinar cells are white with black nucleus. The small circle on the ductal epithelium cell represents expression of hAQP1. After the successful construction of hAQP1, more fluid could be secreted from the duct epithelial cells, and thus more “saliva” could be secreted into the mouth. However, the “saliva” here is different from the saliva secreted by acinar cells in terms of concrete components [129].

After RT, the absence of a large amount of primary fluid and damage to the acinar cells results in the inhibition of the reabsorption of ions from primary saliva by SG ducts, which may lead to an osmotic gradient between the ductal epithelium and fluid in the ducts [129, 130]. In this case, a convenient water channel can be constructed on duct epithelial cells by transferring human aquaporin-1 (hAQP1) cDNA to assist fluid secretion and relieve xerostomia, as shown in Figure 2 [129, 130].

There are two main ways to transfer hAQP1 to SGs in animal models: viral vectors and nonviral vectors [129, 131, 132]. Viral vectors have a higher efficiency than nonviral vectors, but they are more likely to trigger immune rejection in hosts than nonviral vectors [132, 133].

The adenoviral vector is one of the most commonly used vectors in gene therapy, with high transduction efficiency [134]. This vector has been successfully used in clinical trials [129, 135, 136]. Owing to the immune rejection in hosts, a recombinant serotype 5 adenoviral vector-encoding hAQP1, AdhAQP1 can only provide an effective therapeutic outcome for a short time in animal experiments. However, a phase I trial showed that xerostomia was relieved in 5 of the 11 subjects for 2-3 years, and the parotid flow rate remained significantly elevated 3–4.7 years after treatment [135–138]. This may be related to the lack of methylation of the human cytomegalovirus promoter in human SG epithelial cells [139].

Another commonly used viral vector is the serotype 2 adeno-associated viral (AAV2) vector, which triggers milder immune rejection in the host [140]. The results obtained in irradiated miniature pigs suggest that the use of AAV2 as a transduction vector may serve as a way to increase salivary flow over a longer period than AdhAQP1 [140]. The ultrasound-assisted gene transfer method is noteworthy. In animal experiments, ultrasound-assisted gene transfer can generate a “sonoporation” effect to assist gene transfer to SG cells without introducing viral antigens [131, 141]. In experiments to improve SG function in irradiated miniature pigs, this approach achieved a therapeutic efficacy comparable to that of AdhAQP1 and reduced the risk of immune responses in the host triggered by viral vectors [131].

In addition to encoding hAQP1 to improve xerostomia, the prevention of IR-induced SG hypofunction by adenovirus-encoded growth factor and neurotrophic factor deserves further investigation [142–144]. It has been shown that injection of neurturin adenovirus into mouse SGs prior to IR can reduce parasympathetic cell apoptosis, thereby inhibiting IR-induced decline in SG function in mice through acetylcholine signaling, which is essential for SG development and regeneration [144–146].

### 4.6. Stem Cells

**Figure 3:**
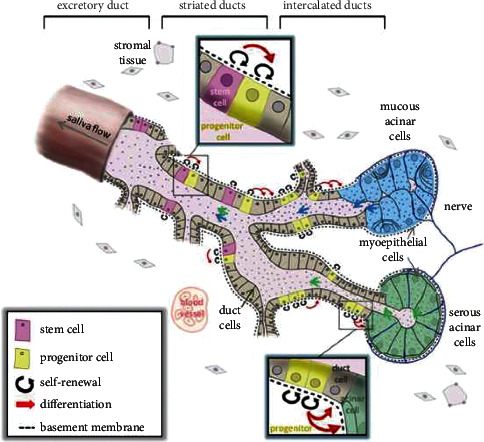
Schematic diagram showing the location, proliferation, and differentiation of stem and progenitor cells in SGs [147].

Stem cells are thought to play a vital role in SG formation and recovery from damage, as shown in Figure 3 [147]. Most of the existing treatment methods involve temporary improvement of xerostomia. However, the application of stem cells in its treatment provides the possibility for the long-term recovery of SG tissue and secretory function [148]. SG stem cells can be collected in advance and implanted after RT. The collection of stem cells often depends on their marker expression profiles. According to known literature, c-kit (CD117) is the most studied marker, CD49F, CD29, CD24, and CD133 also have potential as markers and should be used in combination to improve accuracy [149]. The potential of SG cells to aid their recovery after RT was confirmed by isolating the c-Kit cell population, producing a salisphere *in vitro*, and transplanting it [150, 151]. Salisphere transplantation can not only replace the lost proliferative cells of the SGs post-RT but may also benefit patients' endogenous cells [152]. In addition, SG organoids can be produced using 3D extracellular matrices, which can make cell differentiation similar to that of SGs to obtain a better 3D structure. For clinical applications, SG organoids have been made with biocompatible magnetic nanoparticles [148, 153–155].

Furthermore, the isolated stem cell population that can be used for clinical treatment needs to be assessed. The impact of the patient's age, the number of cells to be transplanted during treatment, the accuracy of transplant cells delivered to the desired site, and confirmation of genomic stability during cell culture to prevent potential cancer cells from being transplanted into the patient are challenges for stem cell applications [147, 156]. Though the role of stem cells in restoring SGs is still unclear, there is a view that SG renewal is affected more by the proliferation of acinar cells [157].

In addition to SG cells, stem cells derived from other parts of the body have also been reported, among which adipose-derived stem cells (ADSCs) are the most promising. Meanwhile, mesenchymal stem cells from the bone marrow, labial mucosa, and dental pulp have also proved valuable in treating xerostomia [148]. It has been suggested that ADSCs can be obtained by non-invasive surgery, and their potential for induction of SGs cells has been confirmed, where this transdifferentiation can be facilitated by platelet-rich fibrin [158, 159]. However, there are studies that did not observe significant transdifferentiation of ADSCs into SG. ADSCs also secrete paracrine factors to maintain amylase secretion. Although the mechanism of action remains controversial, the positive effects of ASCs on SGs are certain [160]. Notably, ASCs were included in phase I/II clinical trials and showed good safety and efficacy [161]. Bone marrow mesenchymal stem cells are also shown to increase the expression of SDF1-CXCR4, Bcl-2, and other proteins after transplantation under hypoxic conditions, thus promoting cell proliferation and differentiation, leading to the recovery of SGs [162]. Injection of labial stem cell extracts improved blood vessel, nerve, and cell recovery in mice, and increased saliva flow rates by 50–60% [163]. Dental pulp stem cells can be easily obtained, and their anti-inflammatory effects, multipotent differentiation properties, and migration to damaged tissues illustrate their potential for application in the field of xerostomia treatment [148, 164]. Although they have potential in the treatment of xerostomia along with SGs cells, issues such as efficacy, preservation, transport methods, and safety remain formidable challenges.

## 5. Conclusion

We found improvements in most diagnostic methods but with shortcomings. Most of the prevention and treatment methods are restricted to animal experiments, requiring further clinical research, where antioxidants, gene transfer, and stem cell transplantation have promising developmental and therapeutic prospects.

## Figures and Tables

**Table 1:** Summary of measurement methods of saliva flow rate.

Type	Methods	Procedure	Pros	Cons
Unstimulated whole saliva [UWS]	Draining method	Allow the saliva to flow naturally down the lower lip and collect it in a graduated container [21]	No effect of slow rate [25] More representative of unstimulated secretion [25] Good repeatability [11] Easy use [11]	Time-consuming [26] Unattractive [26] Remaining saliva in oral [27] Need good collaboration [25]
	Spitting Method	Allow the saliva to cumulate at the bottom of the mouth for a while and then spit into a graduated container [21]	Less evaporation of saliva [25] Good repeatability [11] Easy use [11]	Time-consuming [26] Unattractive [26] Incomplete spit [27] Stimulatory effects [25] Need good collaboration [25]
	Swab method	Allow the saliva to be absorbed with pre-weighed cotton rolls and reweighed at the end of the collection period [21]	Low cost [25] Easy use [25] Suitable for less- or non-collaborative patients [25]	Risk of swallowing [25] Possible stimulatory effect [28]
	Suction method	Allow the saliva to be sucked into a graduated container by a negative pressure suction device in the closed or open suction method [21]	High reliability [29] Time-saving [29] No strict demands for Collaboration [29]	Require skilled personnel [29] Possible stimulatory effect [28]
	BokaFlo™	Place BokaFlo™ disposable device under the subject's tongue to collect saliva, then the device was removed and placed on the BokaFlo™ instrument for measurement [30]	High sensitivity [30] High specificity [30] Comfortable [30] Time-saving [30]	Underestimate saliva flow rate [30]

Stimulated whole saliva [SWS]	Acid stimulation method	Place a solution of 2% citric acid on each side of the tongue every 30 seconds for five minutes and collect saliva [21]	Get more saliva in a short time [31]	Change the composition of saliva [25] Change salivary pH [25] Need collaboration [25]
	Chewing method	Collect saliva after chewing an unflavored gum base or paraffin wax [21]	Get more saliva in a short time [31]	Change the composition of saliva [25] Change salivary pH [25] Uncontrollable chewing force and force duration [32]

Major salivary gland	Parotid gland	Place the Lashley cup at the mouth of the parotid gland catheter to collect saliva from the parotid gland [32]	Not invasive [25]	Time-consuming [33] Complex procedure [33] Required skilled personnel [33]
	Submandibular gland and sublingual gland	Use a Wolff saliva collector to collect saliva from the submandibular and sublingual gland [32]	Not invasive [33]	Time-consuming [33] Complex procedure [33] Require skilled personnel [33]
Minor salivary gland	Iodine-starch filter paper method	Place the iodine-starch filter paper on the lower lip for a period of time and then scan and digitize with an image scanner [34]	Comfortable [34] Easy use [34] Larger area of lower lip [34]	No large volume collected [34] Contain harmful iodine [34] Not for people allergic to iodine [34]
	Electronic sialometry device	Measure the electrical resistance of a filter paper that has absorbed saliva, providing an estimate of saliva volume [35]	Easy use [35] Low cost [35] Quick [34]	No large volume collected [35]
	Standard filter paper	Place a standard filter paper on the buccal mucosa to absorb saliva and weigh it [36]	Not invasive [25] Easy use [36]	No large volume collected [36]

## Data Availability

All data, figures, and tables in this review paper are labeled with references.
